# Supplementary material for: Inferring incompetence from employment status: An audit-like experiment
Source: PLoS One. 2023 Mar 9;18(3):e0280596. doi: 10.1371/journal.pone.0280596 (PMC9997970; doi:10.1371/journal.pone.0280596)
Supplement: Attachment — Submitted filename: PLOS One Revisions_R1.docx [file pone.0280596.s001.docx]

Dear Dr Brito-Costa,

Thank you very much for your positive response in the first round of revision. We were very encouraged by your decision letter and the reviewer’s comments. We explain below how we dealt with the individual points mentioned.

1. Please ensure that your manuscript meets PLOS ONE's style requirements, including those for file naming. The PLOS ONE style templates can be found at
   <https://journals.plos.org/plosone/s/file?id=wjVg/PLOSOne_formatting_sample_main_body.pdf> and 
   <https://journals.plos.org/plosone/s/file?id=ba62/PLOSOne_formatting_sample_title_authors_affiliations.pdf>

We have now updated the document and use the PLOS One style template throughout.

2. Peer review at PLOS ONE is not double-blinded (<https://journals.plos.org/plosone/s/editorial-and-peer-review-process>). For this reason, authors should include in the revised manuscript all the information removed for blind review.

We have now included the title page with all authors identified and unblinded the references.

3. Please amend your current ethics statement to address the following concerns:
   a) Did participants provide their written or verbal informed consent to participate in this study?
   b) If consent was verbal, please explain i) why written consent was not obtained, ii) how you documented participant consent, and iii) whether the ethics committees/IRB approved this consent procedure.

We used an online survey where people read the informed consent before they had to agree/disagree with the survey before they could continue. We have also included information that the LSE ethics committee approved the study.

4. We note that you have stated that you will provide repository information for your data at acceptance. Should your manuscript be accepted for publication, we will hold it until you provide the relevant accession numbers or DOIs necessary to access your data. If you wish to make changes to your Data Availability statement, please describe these changes in your cover letter and we will update your Data Availability statement to reflect the information you provide.

We will be able to provide a DOI number for the data, which is Identifier: **DOI 10.17605/OSF.IO/RF7HW**

We will be able to update the Data Availability statement with this.

5. Please review your reference list to ensure that it is complete and correct. If you have cited papers that have been retracted, please include the rationale for doing so in the manuscript text, or remove these references and replace them with relevant current references. Any changes to the reference list should be mentioned in the rebuttal letter that accompanies your revised manuscript. If you need to cite a retracted article, indicate the article’s retracted status in the References list and also include a citation and full reference for the retraction notice.

 Garcia-Lorenzo, L., Sell-Trujillo, L., & Donnelly, P. (2021). Responding to stigmatization: How to resist and overcome the stigma of unemployment. Organization Studies, 01708406211053217.

- Suttill, B. (2021). Non-academic, lazy and not employable: Exploring stereotypes of NEETs in England. Employment and Careers.

Thank you very much, we have included these in the manuscript; we are not citing any work that was retracted.

6. The second concern relates to the communication strategy followed in the article. For example, although Table 1 and 3 summarize descriptive statistics of each study, they omit third and fourth statistical moments (asymmetry and kurtosis) that might be informative for a general audience if communicated with ridgeplots. Given the fact that authors analyzed their data with R-codes, it would be nice to see statistical differences visually.

Thank you also for this comment; we have now included the information on symmetry and kurtosis in the descriptive tables. We have not included a ridgeplot as we felt is was not necessary to include more Figures.

In sum, given our revision, we hope that you find the revised manuscript to be of wide interest and suitable for publication in *PLoS One* and we look forward to your editorial response.

Sincerely,

Dr Ilka Gleibs as corresponding author on behalf of all authors.
